# Design of LDMOS Device Modeling Method Based on Neural Network

**DOI:** 10.1155/2022/4988636

**Published:** 2022-08-10

**Authors:** Teng Liu, Tianlong Wen, Wentong Zhang, Nailong He, Sen Zhang, Hua Song

**Affiliations:** ^1^Technology Development Department, CSMC Technologies Corporation, Wuxi 214000, China; ^2^State Key Laboratory of Electronic Thin Films and Integrated Devices, University of Electronic Science and Technology of China, Chengdu 610054, China

## Abstract

The rapid development of power semiconductor devices is helping to realize a low-carbon society and provide a better life for everyone. Power semiconductors not only are used in many large-scale industrial control fields such as power transmission and control in power grids, rail transit traction systems, and defense weapons and equipment, but also play a vital role in daily equipment such as home appliances, medical electronics, and electronic communications; all devices such as power steering in cars, battery chargers, cell phones, and microwave ovens utilize power electronics. This research mainly focuses on the high-voltage LDMOS device model and its implementation. Based on the in-depth study of the structure and physical mechanism of high-voltage LDMOS devices, with the help of BSIM4 core model, which is now very mature and widely used in industry, the drift region of high-voltage LDMOS is mainly modeled, and the drift region of LDMOS is modeled as a variable resistance controlled by voltage. Finally, Verilog-A language and neural network method are used to establish a compact model of LDMOS. The improved model is applied to LDMOS and can better fit the output characteristics with self-heating effect.

## 1. Introduction

The 21st century is an era of rapid development of science and technology, especially the rapid progress of electronic information technology, which benefits from the progress of the semiconductor industry and the development of the chip industry. In recent years, the development of our country's integrated circuit industry has a wide variety of integrated circuits and is used in automotive electronics, home appliances, mobile phones, computers, and wearable electronic devices. As the feature size of integrated circuits shrinks, the current state-of-the-art fabrication process has entered the 7 nm era. According to the scaling theory, the operating voltage of Metal-Oxide-Semiconductor Field-Effect Transistor (MOSFET) devices also decreases as the feature size of the device decreases. The operating voltage has dropped below 1 V, but high-voltage devices in power management, display drive, digital media, and mixed digital chips are still necessary [1–5], among which LDMOS (Laterally Diffused MOSFET) device is a high-voltage device used in integrated circuits, and its performance and stability have been widely concerned as its important parameter indicators. At present, many foreign chip manufacturers have set up special departments to extract model parameters. Foreign research on model parameter extraction is relatively early, and the overall semiconductor technology level is relatively high, so the model parameter extraction technology is relatively advanced. In China, the development of model parameter extraction is slow and backward, which has a certain relationship with the overall backwardness of domestic semiconductor technology. In addition, the domestic semiconductor industry does not pay attention to the extraction of model parameters, and there is a relative shortage of talents in this area. Therefore, it is very important to increase the investment in model parameter extraction to improve the overall level of the semiconductor industry. In this study, on the basis of analyzing the characteristics of high-voltage LDMOS, the drift region is modeled, and some special effect models are established at the same time. And the built LDMOS model is implemented by Verilog-A language, and the model parameter extraction and simulation curve fitting are completed. [1–9].

## 2. Related Work

Semiconductor device model is an abstraction that ignores the internal structure of semiconductor devices and directly reflects the output and transfer characteristics of devices. There are many kinds of semiconductor device models, which can be divided into DC, transient, and AC models according to different signal types. DC model is a static model, AC model is a small signal model, and transient model is a large signal dynamic model. According to the requirements of computational efficiency, device models can be divided into analytical model, look-up table model, and empirical model. SPICE model is one of the semiconductor device models. Its biggest feature is that it can be directly used in circuit simulator. In short, spice model is composed of a set of formulas and a set of parameters. Formulas are used to describe the characteristics of semiconductor devices. Parameters are the coefficients in these formulas. The establishment process of these formulas is called “modeling,” which is generally completed by universities or scientific research institutes. The process of extracting model parameters is called “model extraction,” which is usually completed by chip manufacturers, because different processes have corresponding model parameters. Generally speaking, model formulas have been integrated into circuit simulators, such as spice model BSIM4 of MOSFET 3. Therefore, the chip manufacturer only needs to hand over the extracted model parameters to the circuit designer, and the circuit designer can get the spice model of the corresponding process. Spectre is a completely customized integrated circuit simulation tool launched by cadence. It is mainly used for simulation of analog and digital analog hybrid integrated circuits. It is mainly used for UNIX workstations. It solves some numerical problems of spice, is suitable for RF circuit simulation, and supports all Berkeley MOS models. As mentioned above, there are several spice simulators, which must be used together with spice model for circuit simulation. These two together constitute the spice program. Spice simulator is spice's math engine tool. It consists of some basic subroutines for mathematical analysis. One of the typical subroutines is to solve a matrix composed of *n* linear equations. When combined with Newton Raphson iteration, they can also solve the nonlinear equations of static node voltage and branch current of a given circuit. Another basic subroutine is to solve differential equations, which usually requires time as a variable for transient analysis. In these transient analyses, both voltage and current are functions of time. The second part of the spice program is the device model. There are many semiconductor devices in a circuit, such as diodes, bipolar transistors, capacitors, and MOS; these devices correspond to the device model [10–14]. In this study, the structure and physical characteristics of LDMOS devices are analyzed. On the basis of physical derivation, an approximate method is used to establish a semiempirical compact model.

## 3. Related Theoretical Methods

### 3.1. The Basic Principle of WGAN

The original GAN is accompanied by problems such as gradient disappearance and model collapse. When dealing with some problems in specific fields, if it is not optimized, it will not get the desired results. In recent years, GAN has developed rapidly. Various optimization models of GAN have appeared one after another, and there are more and more derivative networks based on GAN. These problems have been solved after the emergence of WGAN. The original GAN used the KL divergence and the JS divergence to measure the distribution of two probabilities, but using such a measure presents a problem if the generated data and the real data are far apart and they do not overlap or overlap. When it can be ignored, the value of JS is constant, which means that the gradient is zero, the gradient disappears, and the training cannot proceed. Therefore, WGAN uses the Wasserstein distance to measure the distribution of two probabilities, and the expression of the Wasserstein distance is as follows [15, 16]:

(1) $$\begin{array}{c} W\left(P_{\text{data}},P_{g}\right)=\underset{a\in \prod \left(P_{\text{data}},P_{g}\right)}{\text{inf}}E_{\left(x,y\right)-a}\left[\left\|x-y\right\|\right]. \end{array}$$

P_data_ represents the real data, *P*_g_ represents the generated data, ∏(*P*_data_, *P*_*g*_) represents the set of joint probability distributions of the real data and the generated data, and the marginal probability distributions of the joint probability distribution are P_data_ and Pg. (x, y) is any one sampled from the set of joint probability distributions; it represents how much weight from the *P*_g_ distribution to the P_data_ distribution is transported from *x* to *y*, so the Wasserstein distance is actually a function of calculating the optimal transport cost. The advantage of Wasserstein distance and JS distance is that even if the two distributions do not overlap or overlap very little, they can still distinguish the distance between the two distributions and keep the gradient updated continuously. Whereas the JS divergence is constant in this case, the gradient will vanish, the model will crash, and the KL divergence may be meaningless. The emergence of Wasserstein distance solves the problem of gradient disappearance and model collapse during original GAN training. It mainly uses the Lipschitz function to limit the range of gradients and avoid extreme conditions of gradients, thereby ensuring smooth training of GANs. To express the capabilities of the Lipschitz function, convert the Wasserstein distance to the formula:

The definition of the Lipschitz limit is also very simple; that is, for a continuous function, there is a K greater than or equal to zero, so that the maximum value of the absolute value of the derivative of a certain interval in the definition domain is less than or equal to K, then K can be called the Lipschitz constant, and the continuous function is called the Lipschitz constant. A function is a function with a K-Lipschitz condition, and the expression for the K-Lipschitz condition is as follows:

(2) $$\begin{array}{c} \left|f\left(x_{1}\right)-f\left(x_{2}\right)\right|\leq K\left|x_{1}-x_{2}\right|. \end{array}$$

At this time, the loss function of WGAN can be expressed as

(3) $$\begin{array}{c} V\left(G,D\right)=\underset{G}{\min}\underset{D\in K-\text{Lip}}{\max}\left\{E_{x-P_{\text{data}}}\left[D\left(x\right)\right]-E_{x-P_{G}}\left[D\left(x\right)\right]\right). \end{array}$$

There are some requirements for the discriminator; it must be a smooth function, as long as the value of K in the discriminator is not positive infinity, because it will only increase the gradient by K times and will not change the direction of the gradient. For the processing of gradients, a simple processing method can be adopted to limit the weight parameters of the neural network in the discriminator to a certain range [−c, c]; that is to say, when the parameters are updated, for the parameters less than −c, the weight is set to −c, and the weight greater than *c* is set to *c*, so the gradient will not exceed a certain range, which just satisfies the Lipschitz condition. In actual experiments, the weight parameters are updated first, and then the weights are clipped back to this range. The training process of WGAN is as follows:

First initialize a discriminator parameter *w* and generator parameter, initialize a clipping constant *c*, and optimize *D* first in each round of iterative training:

1. Sampling a batch of data 1 2 (x^1^, x^2^,…,x^m^) from the real dataset.
2. Sample a batch of random noise 1 2 (*Z*^1^, *Z*^2^,…, *Z*^3^) from the prior random distribution.

WGAN has also made some changes in network design from the original GAN:

① Do not take the logarithm of the loss in the generator and discriminator.
② Each time the parameters of the discriminator are updated, their absolute values are truncated to a fixed range.
③ The last layer of the discriminator removes the sigmoid function.
④ Do not use momentum-based optimization algorithm (momentum Adam); use RMSprop (root mean square prop) or SGD (stochastic gradient descent).

### 3.2. Structure and Process Method of LDMOS

#### 3.2.1. Structure and Process of LDMOS

**Figure 1:**
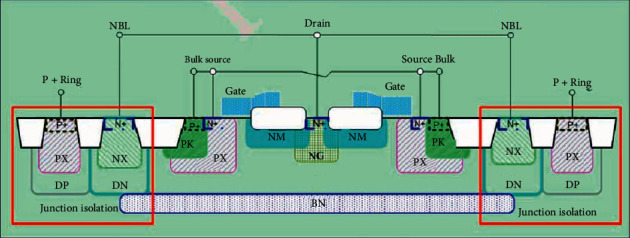
25 V NLDMOS structure cross-sectional view.

This study takes the NLDMOS of the CSMC 0.18 um 25 V BCD process platform as an example to illustrate the structure and process flow of the LDMOS.  Figure 1 shows a cross-sectional view of the LDMOS structure. This is an LDMOS with a bilateral structure. Two identical LDMOS devices are connected in parallel, which can improve the current capability of the LDMOS without changing other characteristics of the device. The main difference between a basic LDMOS device and ordinary MOS in structure is as follows: first, LDMOS is an asymmetric structure. In order to improve the withstand voltage, a relatively long and low doping concentration will be formed on the side of the device near the drain. The drift region can effectively reduce the parasitic capacitance between the source and drain and weaken the short channel effect of LDMOS; secondly, the channel of LDMOS is formed by the lateral double diffusion process, that is, the P-type and the diffusion of N-type impurities, so the channel region is nonuniformly doped, and the formation of the channel is not determined by the gate length. The double diffusion process can precisely control the length of the channel and make the channel length very short. Compared with ordinary MOS, the current capability, transconductance, maximum operating frequency, and speed are greatly improved. Finally, the polysilicon gate of LDMOS not only covers the gate oxide above the channel region, but also covers the gate oxide and field oxide above the drift region. Because the thickness of the field oxide is much larger than that of the gate oxide, the polysilicon is removed from the gate. There is a slope from the top of the oxygen to the top of the field oxygen. In fact, the effective gate length is shorter than the physical length of the gate. [17–19].

For the specific LDMOS of 0.18 um 25 V BCD process, its structural levels mainly include P-type substrate (Psub), N-type buried layer (BN), deep P-well (DP), deep N-well (DN), high-voltage P-well (PX), high-voltage N-well (NX), P-sinker (PK), P-shift (PM), N-shift (NM), N-offset (NG), *N* + Source/Drain (*N*^+^), *P*^+^ body contact area, surface field oxide layer, polysilicon gate (Gate), source and drain metal electrodes, P substrate electrodes, etc.

**Figure 2:**
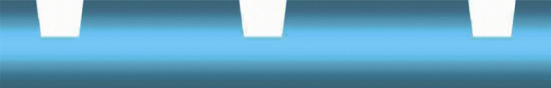
ZO loop process.

As shown in Figure 2, first generate 1000 A TEOS, and then perform ZO lithography to form part of the stripe pattern. The ZO cycle process is only done in the dicing lane area, and there is no ZO pattern in the device area. This process is beneficial to the alignment of the next BN, DN, DP, and PM layers.

**Figure 3:**
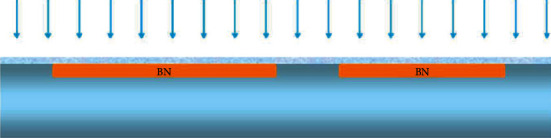
BN and BP process.

Then there are N buried layer and P buried layer process, as shown in Figure 3. First perform BN implantation and push well to form an N-type buried layer. This process has a good isolation effect and can also effectively prevent the upper and lower punch-through breakdown of PLDMOS in the same BCD process. The trap is then pushed in nitrogen, followed by a field oxygen of 3000 A to eliminate defects. Next is the fabrication of the P buried layer (BP). Figure 3 and the following schematic diagrams omit the BP structure. The formation of BP structure can effectively improve the punch-through breakdown between BNs.

**Figure 4:**
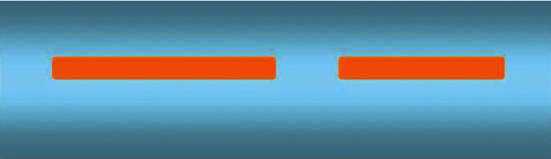
EPI loop process.

Next is the epitaxy process, forming a P-type epitaxy layer with a thickness of 4.7 um, as shown in Figure 4.

**Figure 5:**
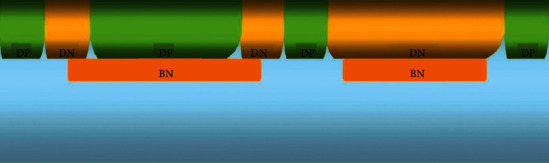
DN and DP loop process.

There are various isolation technologies in the BCD process. Here, a Deep N-well (DN) and a Deep P-well (DP) are formed successively to form a PN junction with isolation. The DN and DP used to make LDMOS devices are also here, formed simultaneously in one step, as shown in Figure 5.

**Figure 6:**
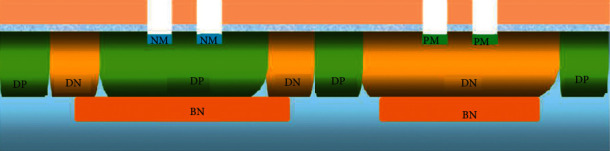
PM and NM loop process.

In order to improve the characteristics of the LDMOS drift region, it is necessary to process the drift region and perform P-shift (PM) and N-shift (NM) implantation in the drift regions of PLDMOS and NLDMOS, respectively, as shown in Figure 6.

**Figure 7:**
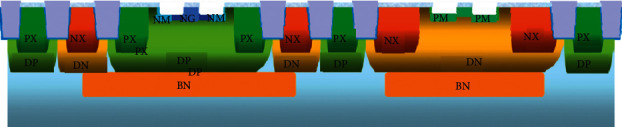
5 V N/P-well.

In the process of making high-voltage LDMOS, a 5 V N/P-well process is sometimes inserted, as shown in Figure 7. This process sequentially performs high-voltage N-well lithography, high-voltage N-well implantation, high-voltage P-well lithography, and high-voltage P-well implantation. This process can be used to adjust the threshold voltage Vt of high-voltage LDMOS.

#### 3.2.2. Special Effects of LDMOS Devices

LDMOS is a MOS device with a special structure. When applied to low voltage, it has the characteristics of general MOS devices. For example, when Vds = 0.1 V, the subthreshold region characteristics and transfer characteristics of LDMOS are similar to those of ordinary MOS. However, LDMOS devices are usually designed for high-voltage applications, which determines their device characteristics that are different from ordinary MOS devices. Due to the application of high voltage, the high electric field inside the LDMOS device makes it exhibit some special effects, such as quasi-saturation effect, self-heating effect, and impact ionization effect. In fact, some of these special effects also appear in low-voltage MOSFETs, such as self-heating effects and impact ionization, because the electric field strength in low-voltage MOSFETs can become quite large as the channel length decreases [20–22].

## 4. LDMOS Modeling

### 4.1. General Drift Region Modeling

This research mainly studies the drift region modeling of LDMOS based on the BSIM4 model, taking a 0.18 um 25 V BCD process platform as an example. The LDMOS drift region is equivalent to a voltage-controlled resistor controlled by gate voltage and drain voltage. Using a voltage-controlled resistor to model the LDMOS drift region, the model is relatively simple, which can not only ensure high accuracy, but also make the calculation converge quickly; by considering the depletion of the N-well in the LDMOS drift region, the JFET effect is formed, and a JFET is used, with device and a drift region resistor to model the drift region. An equivalent circuit is used to model the LDMOS drift region.

**Figure 8:**
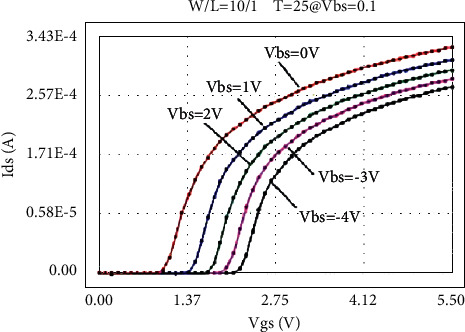
Transfer characteristic at Vds = 0.1 V.

**Figure 9:**
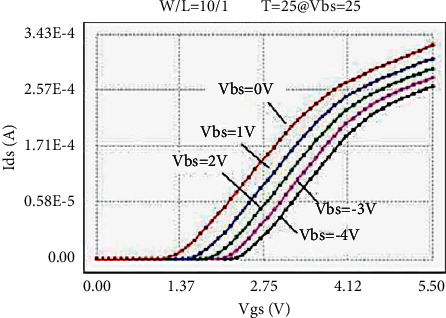
Transfer characteristic at Vds = 25 V.

**Figure 10:**
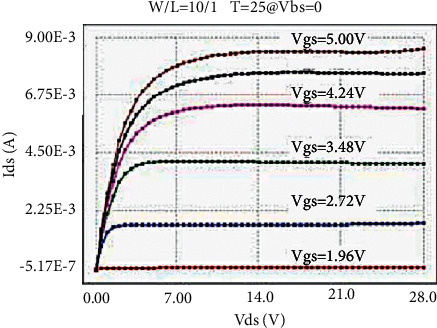
Output characteristics at Vbs = 0 V.

**Figure 11:**
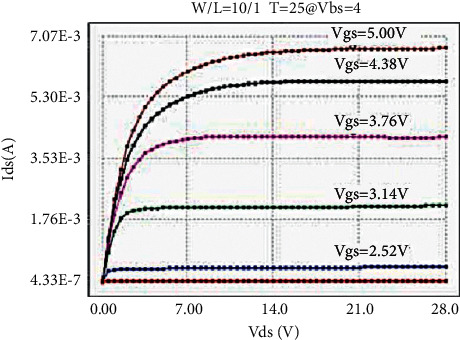
Output characteristics at Vbs = −4 V.

The LDMOS is tested for electrical characteristics, mainly to test the transfer characteristics and output characteristics. The test conditions are as follows: when the drain voltage Vds is 0.1 V and 25 V, respectively, the gate voltage Vgs step size is 0.1 V, and the maximum voltage is 5.5 V for transfer characteristics; when the substrate bias Vbs is 0 V and −4 V, respectively, there are output characteristics with a drain voltage step size of 0.5 V and a maximum voltage of 28 V at V. The test results are as follows: the threshold voltage Vt is 0.95 V, and the operating voltage ion is 8.46 mA. The curves obtained from the test are as follows: the transfer characteristic curve at Vds = 0.1 V as shown in Figure 8, the transfer characteristic curve at Vds = 25 V as shown in Figure 9, and the transfer characteristic curve at Vbs = 0 V as shown in Figure 10. The output characteristic curve and the output characteristic curve when Vbs = −4 V are shown in Figure 11.

**Figure 12:**
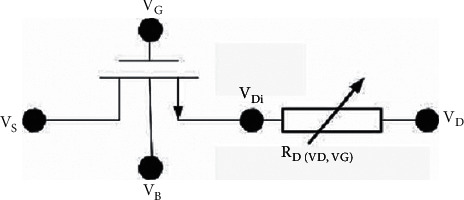
LDMOS equivalent circuit diagram.

When the external bias is small, the resistance of the channel region accounts for the main part, and the resistance of the drift region is less affected by the external bias, which can be regarded as a constant resistance. When the device is applied to a high-voltage bias, the current channel of the drift region becomes narrow, and the resistance increases rapidly, which is much larger than the resistance of the channel region. At this time, most of the voltage drop falls on the drift region. It can be seen that, in the whole working area, the voltage at the node Di in Figure 12 is always kept at a low value, and the internal MOS with Di as the intrinsic drain is little affected by the external high-voltage bias. Therefore, the method of subregional modeling can be used to separate the channel region and the drift region and establish the equivalent circuit diagram as shown in Figure 12. The channel region model still uses the original BSIM4 model, only the drift region needs to be modeled, and the two together constitute the LDMOS model.

### 4.2. Modeling of Special Effects

Due to the special structure and application in high-voltage applications, high-voltage LDMOS devices exhibit some special effects different from ordinary MOSFET devices, such as quasi-saturation effect and self-heating effect. In order to accurately describe the electrical characteristics of LDMOS, it is necessary to establish appropriate models for these special effects.

#### 4.2.1. Quasi-Saturation Effect Model

**Figure 13:**
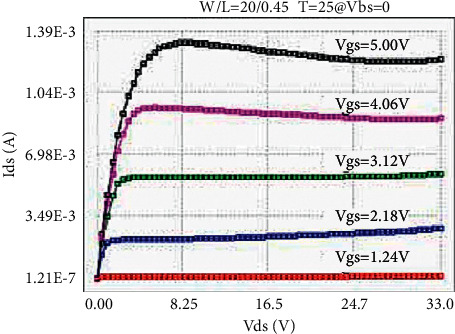
30 V LDMOS output characteristics.

LDMOS devices usually operate at high voltages, and the quasi-saturation effect is one of its common special effects. As shown in Figure 13, when the gate voltage is relatively large, if the drain voltage is relatively small, the LDMOS device is in the linear region, and the drain current increases linearly with the drain voltage; and when the drain voltage is very large, the drain current basically does not change with the drain voltage; that is, it enters the saturation region. Between the linear region and the saturation region, there is a transition region, and the drain current increases slowly with the drain voltage, which is the quasi-saturation region. This is due to the fact that the intrinsic MOS is not yet saturated, and the drift region carriers are the first to reach velocity saturation. In the quasi-saturation region, the intrinsic MOS is in the linear region and the drift region reaches speed saturation. The increase of the gate voltage Vgs will not significantly increase the drain current. At this time, the influence of the gate bias on the drain current is relatively small. Therefore, a voltage-controlled resistor controlled by the gate and drain voltages can be used to model the quasi-saturation effect.

#### 4.2.2. Self-Heating Effect Model

Self-heating effect refers to the effect that the temperature of the device itself increases due to the heat generated by the power loss when the LDMOS device operates at high voltage and high current. Self-heating effects generally appear in devices operating at high power, and the increase in device temperature will affect its electrical properties, such as threshold voltage, mobility, and velocity saturation effects. Self-heating effect is a relatively common physical phenomenon in LDMOS devices. As shown in Figure 13, it is the output characteristic curve of a 30 V LDMOS. Five different gate voltages were tested, the offset was 0, and the maximum drain voltage Vds was measured to be 1.1 times its operating voltage. It can be seen from the figure that when the gate voltage is small or medium, there is no obvious difference from the output characteristics of ordinary MOS. However, when Vgs is greater than 4 V, in the saturation region, with the increase of Vds, the drain current gradually decreases, and an obvious self-heating effect appears. Therefore, it is necessary to consider establishing the self-heating effect model of LDMOS.

## 5. Implementation of LDMOS Model Based on WGAN

The LDMOS model established in this study is relatively independent of its intrinsic MOS part and drift region part, and the Verilog-A language model of BSIM4 has been developed. Therefore, some changes can be made on the basis of the BSIM4 model to complete the modeling of LDMOS.

### 5.1. Drift Zone Model

Since the LDMOS channel region and the drift region are modeled separately, the drift region model has little effect on the channel region model. In order to make the model more extensible, a resistor can be added between the internal node (Si) and the external node (S) of the source. It is very helpful for the realization of the model. In addition, there is a resistor controlled by the gate voltage between the internal and external nodes of the source and drain terminals in BSIM4, which has little effect on the resistance of the drift region and can correct the model and retain it. Based on this, the Verilog-A model of BSIM4 is modified as follows: Define the input parameters. Define the model parameters that need to be tuned, such as

- parameter real rcdw0 = 0;
- parameter real rcsw0 = 0;
- parameter real crd = 0;
- parameter real crs = 0;

#### 5.1.1. Define Variables

- Real rd, rs.
- Real BSIM4rcdw0, BSIM4rcsw0, BSIM4crd, BSIM4crs.

#### 5.1.2. Establish the Drift Region Resistance Model: Taking the Drain Side as an Example, the Source Side Is Similar

- rd_tfac = 1 + BSIM4tcrd1 ^*∗*^ delTemp + BSIM4tcrd2 ^*∗*^ delTemp ^*∗*^ delTemp; *T*0 = pow ((BSIM4crd + BSIM4wcrd/w) ^*∗*^ V (d, di), BSIM4eta);
- *T*1 = BSIM4prwbd ^*∗*^ V (s, b);
- *T*2 = 1/(1 + BSIM4prwgd ^*∗*^ abs (V (g, s))); *T*3 = pow (weff ^*∗*^ 1E6, BSIM4wrd); rd = BSIM4rcdw0 ^*∗*^ (T0 ^*∗*^ (*T*1 + T2) + 1)/T3 ^*∗*^ rd_tfac; Rd = Rd + rd.

#### 5.1.3. Branch Current Contribution

The effect of drift region resistance on drain current is achieved by the following expression: Iddi = V(d, di) ^*∗*^ (BSIM4drainConductance/(1 +  Rd ^*∗*^ BSIM4drainConductance)); I (d, di) <+ Iddi; so far, the more important and difficult part of the LDMOS model, the drift region model, has been modified. Next, the model parameter extraction software MBP can be used to extract parameters from the built VA model. Whether the device model is accurate or not not only depends on whether the model itself is accurate enough, but also is closely related to the extraction of model parameters. In fact, if the device model is very accurate, but the extracted model parameters are not accurate enough; the simulation results often have large errors with the measured data. It can be seen that accurate model parameter extraction is very important for the device model.

**Figure 14:**
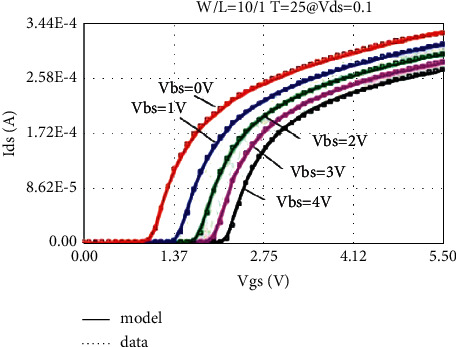
Transfer characteristics of BSIM4 model fitting (Vds = 0.1 V).

**Figure 15:**
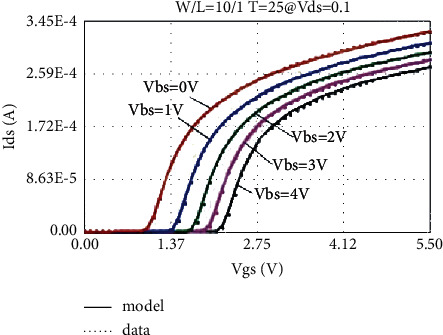
Transfer characteristics fitted by the LDMOS model (Vds = 0.1 V).

Through the above method, the transfer characteristic curves of Vds = 0.1 V at normal temperature adjusted by the original BSIM4 model and the new LDMOS model are shown in Figure 14 and Figure 15, respectively.

By comparing the simulation results of the transfer characteristics of the two models after adjustment, it can be found that, at a lower drain voltage bias, both the BSIM4 model and the LDMOS model can achieve better results.

### 5.2. Self-Heating Effect Model

**Figure 16:**
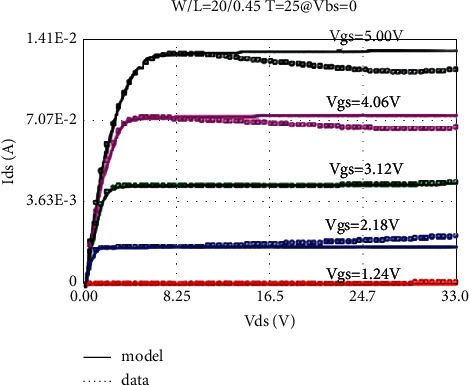
Output characteristics without considering self-heating effect (Vbs = 0 V).

Self-heating effect is a special effect of high-voltage devices, which makes the saturation region of the output characteristic curve of LDMOS devices working in high-voltage state not straight, but slightly downward; that is, a negative resistance phenomenon occurs. Here, a 30 V NLDMOS is used as an example to illustrate the realization of the self-heating effect model. The curve fitting of the BSIM4 model without considering the self-heating effect is shown in Figure 16. Compared with the measured data (dotted line), the model curve (solid line) has a relatively large deviation, and the trend of output current decline cannot be fitted.

The self-heating effect is achieved by contributing the self-heating effect to the drain current in the form of a drain current drop. The specific implementation method is as follows.

#### 5.2.1. Definition of Parameters and Variables

- Parameter real Kth = ‘NOT_GIVEN; parameter real Rth0 = ‘NOT_GIVEN.
- Parameter real Rti = ‘NOT_GIVEN.
- Real BSIM4Kth, BSIM4Rth0, BSIM4Rti, vdese.
- Integer BSIM4KthGiven, BSIM4Rth0Given.

  5.2.2. Assign Values to Intermediate Variables

- BSIM4Kth = Kth.
- BSIM4Rth0 = Rth0.
- BSIM4Rti = Rti.

  5.2.3. If the Parameter Is Not Initialized, It Needs to Be Given a Default Value

- BSIM4KthG iven =  (BSIM4Kth = = ‘NOT_GIVEN) ? 0 : 1.
- BSIM4Rth0Given =  (BSIM4Rth0 = = ‘NOT_GIVEN)? 0 : 1.
- BSIM4RtiG iven =  (BSIM4Rti = = ‘NOT_GIVEN)? 0 : 1.
- If (!BSIM4KthGiven).
- BSIM4Kth = 1.
- If ( !BSIM4Rth0Given) BSIM4Rth0 = 1.
- If (!BSIM4RtiGiven) BSIM4Rti = 1.

#### 5.2.2. Establish a Self-Heating Effect Model

- vdese = abs (V (s, si)) + abs (V (di, si)) + abs (V (d, di));
- *T*0 = BSIM4Rti ^*∗*^ cdrain ^*∗*^ Tnom; *T*1 = T0 ^*∗*^ vdese; *T*2 = Tnom + *T*1 ^*∗*^ BSIM4Kth;
- *T*3 = BSIM4Rth0—BSIM4Kth ^*∗*^ Tnom; cdrain = 2 ^*∗*^ T0/(*T*2 + sqrt (T2 ^*∗*^*T*2 + 4 ^*∗*^ T1 ^*∗*^ T3)).

**Figure 17:**
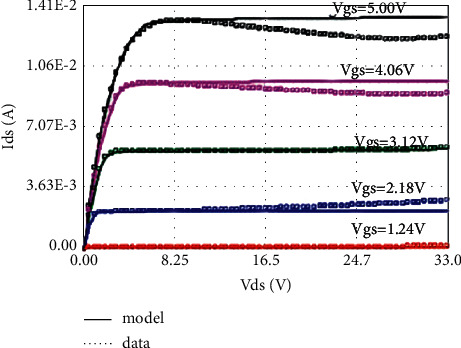
Output characteristics considering self-heating effect (Vbs = 0 V).

So far, the LDMOS self-heating effect model has been established. The self-heating effect model has few parameters, only three parameters Kth, Rti, and Rth0. The extraction of the parameters of the self-heating effect model is also very simple. First, fit the output characteristic curve without considering the self-heating effect and then adjust the three parameters of Kth, Rti, and Rth0 to make the output characteristic curve slope downward. Of course, this process may also need to be repeated several times. The adjusted output characteristic curve with self-heating effect is shown in Figure 17. By comparing with Figure 16, it can be found that the self-heating effect part of the model has been significantly improved.

## 6. Conclusion

LDMOS is an important high-voltage device in BCD process. It has been widely used because of its high-voltage resistance and strong driving ability. Therefore, it is particularly important to establish an accurate LDMOS model. Based on the LDMOS physical model established by theoretical analysis, this research rewrites the Verilog-A language model code and related neural network of BSIM4 to realize the LDMOS model. The first is to implement the drift region model. After defining the parameters and variables, a voltage-controlled resistor controlled by the gate voltage and the drain voltage is added to the original drain resistor, and the temperature effect is considered and optimized. The model fits well the output characteristic curve of a 25 V LDMOS at room temperature and the characteristic curves at different temperatures. For the self-heating effect, the thermal resistance and heat-capacitance network model is simplified, and only the influence of thermal resistance on the channel current is considered. The improved model is applied to a 30 V LDMOS, and the output with self-heating effect is well fitted. Although this paper implements the Verilog-A language modeling of LDMOS in general, there are still some deficiencies. Because the original BSIM4 model is used in the dynamic characteristic model, the gate drain capacitance without considering the drift zone capacitance is not accurate enough.

## Data Availability

The dataset can be accessed upon request.
